# Improving Detection of Disease Re-emergence Using a Web-Based Tool (RED Alert): Design and Case Analysis Study

**DOI:** 10.2196/24132

**Published:** 2021-01-07

**Authors:** Nidhi Parikh, Ashlynn R Daughton, William Earl Rosenberger, Derek Jacob Aberle, Maneesha Elizabeth Chitanvis, Forest Michael Altherr, Nileena Velappan, Geoffrey Fairchild, Alina Deshpande

**Affiliations:** 1 Information Systems and Modeling Group Los Alamos National Laboratory Los Alamos, NM United States; 2 Neutron Science and Technology Group Los Alamos National Laboratory Los Alamos, NM United States; 3 Weill Cornell Medical Cornell University New York, NY United States; 4 BioFire Diagnostics, LLC Salt Lake City, UT United States; 5 Biosecurity and Public Health Group Los Alamos National Laboratory Los Alamos, NM United States

**Keywords:** disease re-emergence, infectious disease, supervised learning, random forest, visual analytics, surveillance

## Abstract

**Background:**

Currently, the identification of infectious disease re-emergence is performed without describing specific quantitative criteria that can be used to identify re-emergence events consistently. This practice may lead to ineffective mitigation. In addition, identification of factors contributing to local disease re-emergence and assessment of global disease re-emergence require access to data about disease incidence and a large number of factors at the local level for the entire world. This paper presents Re-emerging Disease Alert (RED Alert), a web-based tool designed to help public health officials detect and understand infectious disease re-emergence.

**Objective:**

Our objective is to bring together a variety of disease-related data and analytics needed to help public health analysts answer the following 3 primary questions for detecting and understanding disease re-emergence: Is there a potential disease re-emergence at the local (country) level? What are the potential contributing factors for this re-emergence? Is there a potential for global re-emergence?

**Methods:**

We collected and cleaned disease-related data (eg, case counts, vaccination rates, and indicators related to disease transmission) from several data sources including the World Health Organization (WHO), Pan American Health Organization (PAHO), World Bank, and Gideon. We combined these data with machine learning and visual analytics into a tool called RED Alert to detect re-emergence for the following 4 diseases: measles, cholera, dengue, and yellow fever. We evaluated the performance of the machine learning models for re-emergence detection and reviewed the output of the tool through a number of case studies.

**Results:**

Our supervised learning models were able to identify 82%-90% of the local re-emergence events, although with 18%-31% (except 46% for dengue) false positives. This is consistent with our goal of identifying all possible re-emergences while allowing some false positives. The review of the web-based tool through case studies showed that local re-emergence detection was possible and that the tool provided actionable information about potential factors contributing to the local disease re-emergence and trends in global disease re-emergence.

**Conclusions:**

To the best of our knowledge, this is the first tool that focuses specifically on disease re-emergence and addresses the important challenges mentioned above.

## Introduction

Infectious diseases remain a leading cause of death, contributing to millions of deaths each year [1]. The current COVID-19 pandemic demonstrates the speed with which an infectious disease can travel from one location to another including new locations, and in turn become a global health threat in today’s world of increased travel and globalization. COVID-19 is an infectious disease caused by a newly discovered coronavirus called SARS-CoV-2. In addition to such newly emerging diseases, some diseases that were considered controlled or eliminated are also re-emerging. The past few decades have seen the re-emergence of dengue in Brazil [2], measles in France [3], and yellow fever in Angola [4]. A re-emerging infectious disease is a disease that was a major health problem historically in a location, saw a persistent decline in its incidence, and then saw its incidence increase again. Many factors such as ecological disruptions, changing environment, urbanization and human behaviors, international travel and commerce, and war and civil unrest contribute to the re-emergence of infectious diseases [2,5-8].

Early detection and understanding of disease re-emergence is important for better response and mitigation of these events. However, there are several challenges: The definition of disease re-emergence merely suggests an up–down–up incidence pattern and does not offer any guidance on quantitative measures by which such patterns can consistently identify re-emergence. The current practice of identifying disease re-emergence relies on the knowledge and experience of public health analysts rather than specific criteria, which can lead to inconsistent identification of re-emergence [9]. While high-level factors (such as those mentioned above) contribute to the re-emergence of infectious diseases, it is difficult to identify specific factors contributing to a local disease re-emergence and requires a systematic analysis of a number of factors. Local public health analysts may not have this kind of information readily available. Currently, the recognition and understanding of global disease re-emergence relies on analysis of data about historical outbreaks at the country level around the world [10-13]. Again, such data may not be easily available for the entire world and even if available, retrospective analysis is a time-consuming process. Better methods and data are thus essential to address this challenge.

In the last few years, a number of web-based analytics, tools, and databases have been developed to collect data from multiple sources to monitor disease-related activities [14-16], provide situational awareness [17], or now-cast infectious diseases [18]. While there are currently no tools focused on detecting re-emergence, this presents an opportunity for developing new analytics.

Machine learning algorithms use observation data to identify trends and patterns that can help make better decisions. Supervised algorithms identify patterns from the data that are useful in predicting specific outcomes while unsupervised algorithms extract trends and patterns from the data without relating them to any outcomes. Both supervised and unsupervised methods are used extensively in public health. Unsupervised machine learning is used to understand spatial dynamics of an epidemic [19], extract meaningful structure in electronic health records [20], and identify subgroups among home health patients with heart failure [21]. Supervised machine learning is used for disease forecasting [22,23], mortality risk score prediction in an elderly population [24], predicting blood pressure based on health behaviors [25], and assessing vaccination sentiments [25,26]. Recently, our team developed supervised machine learning models to detect potential infectious disease re-emergence for 4 infectious diseases: measles, cholera, yellow fever, and dengue [9]. Combining such an algorithm with visual analytics could provide a rapid, easy to use, and easy to interpret tool for detecting potential re-emergence.

Visual analysis is a technique that utilizes interactive visualizations to support analytical reasoning [27]. It can help with investigative analysis and hypothesis generation [28] and is especially useful for analyzing large data sets by reducing the load on working memory, offering cognitive support, and utilizing the power of human perception [29]. Recently, visual analytics are increasingly used to analyze data in public health and health care, including human emergency room and veterinary hospital data [30]; relationships between chronic conditions, demographics, behavioral and metal health, preventative health, overarching conditions [31]; and tracking symptom evolution during disease progression [32]. We have also developed a web-based visual analytic for the investigation of infectious disease outbreaks [17].

This paper details Re-emerging Infectious Disease Alert (RED Alert), a web-based tool [33] that integrates our supervised machine learning models [9] with visual analytics to help detect/warn and understand potential re-emergence at both local and global levels for 4 diseases: measles, cholera, dengue, and yellow fever. The diseases were selected in consultation with subject matter experts (SMEs) at the World Health Organization (WHO) as diseases of concern for re-emergence. These diseases also show diversity in transmission and disease burden, allowing us to show transferability of our approach. RED Alert combines disease-related data and analytics needed to help the public health community answer the following questions for detecting and understanding disease re-emergence: Is there a potential disease re-emergence at the local (country) level? What are the potential contributing factors for this re-emergence? Is there a potential for global re-emergence?

This publication describes the methods used to answer these questions and evaluation of machine learning classifiers to detect disease re-emergence and the tool through case studies.

## Methods

### Data

Historical case count data, together with disease subcategories such as severe dengue and deaths, were obtained from the WHO [34-36], Gideon [37], and the Pan American Health Organization (PAHO) [38]. Population data were obtained from 2 data sets: LandScan [39] and the World Bank population data [40]. Rates for measles-containing vaccine first dose and second dose were obtained from the WHO [41] together with the WHO region membership information for each country [42]. The host, pathogen, and environment represent the traditional epidemiological triad [43] and can provide information about the potential causes of re-emergence. For indicators that can be a proxy for re-emergence causes, public health indicator data were obtained from the World Bank [44] using their application programming interface (API) [45]. Detailed information about these data sources can be found in Multimedia Appendix 2.

### Development of RED Alert

RED Alert was developed for application to 4 primary diseases of concern: cholera, measles, dengue, and yellow fever. The visual analytic was developed to have a web application as a front end to the data and analysis. A web API was developed to be used by any program to access the analysis results and underlying data. The back end was developed as a Django-based application. The front end uses JavaScript to read from these API endpoints and dynamically build the corresponding visualizations.

#### Detection of Potential Disease Re-emergence

We integrated previously developed supervised machine learning classifiers to detect potential disease re-emergence for a given location and year [9] into RED Alert. Classifiers are supervised learning algorithms that use a set of labeled data (known observation–class pairs, eg, samples of re-emergence and non-re-emergence events [or outbreaks]) and extract patterns that help predict class (eg, re-emergence or not). These patterns can then be used to map a new observation (eg, outbreak) to a class (eg, re-emergence or not re-emergence).

We used yearly disease data at the country level to train disease-specific classifiers for the 4 diseases: measles, cholera, dengue, and yellow fever. For creating the labeled data set for each disease, the SMEs in our team were given data for 100 countries selected at random (and anonymized), and they labeled each location–year pair as a re-emergence or not. A systematic approach was followed to label the training data. For each disease, SMEs developed a re-emergence schema described in detail by Chitanvis et al [9] that takes into account general disease incidence and trend information (eg, raw incidence, case counts, change in incidence from last few years, or percentile rank) and relevant disease-specific information (eg, vaccination coverage for measles and information on severe dengue cases and death due to dengue) that can help detect potential re-emergence. These factors were organized in a decision tree format to guide the labeling process.

##### Selection of the Classifier

We compared 2 classifiers, decision tree and random forest, using scikit-learn, a free machine learning Python library [46]. See tables 1a-b in [9] for features used for training the classifiers and imputation methods for missing data. For both decision tree and random forest, we explored the following parameter values: (1) Split criteria: gini and entropy; (2) The number of minimum samples required at leaf nodes: 1 to 10; and (3) The number of trees for random forest: 20 to 100.

Precision, recall, and F_1_ are widely used metrics to evaluate the performance of classification and can be calculated as follows:

Precision = True positives/(True positives + False positives)

Recall = True positives/(True positives + False negatives)

F_1_ = 2 × (Precision × Recall)/(Precision + Recall)

As our goal was to identify all potential cases of disease re-emergence while allowing some false positives, we used F_2_ to evaluate the performance of the classifiers. F_2_ takes into account both precision and recall but recall is given more weightage. It can be calculated as follows:

F_2_ = 5 × (Precision × Recall)/([4 × Precision] + Recall)

We evaluated classifiers on held-out test data using nested cross-validation [47], where the inner cross-validation is used to choose the optimal parameters, and the outer cross-validation is used to evaluate the performance of the model with the optimal parameters on a held-out data set to test for overfitting or generalization error. Overfitting occurs when the model learns the structure of the given data set instead of the underlying data-generating phenomenon, so it performs well on the given training data set but fails to perform well on additional data or new observations. We used leave-one-out or 1000-fold cross-validation (whichever is lower) for the inner cross-validation and 10-fold cross-validation for the outer cross-validation.

#### Identifying Potential Contributing Factors for Re-emergence

**Figure 1:**
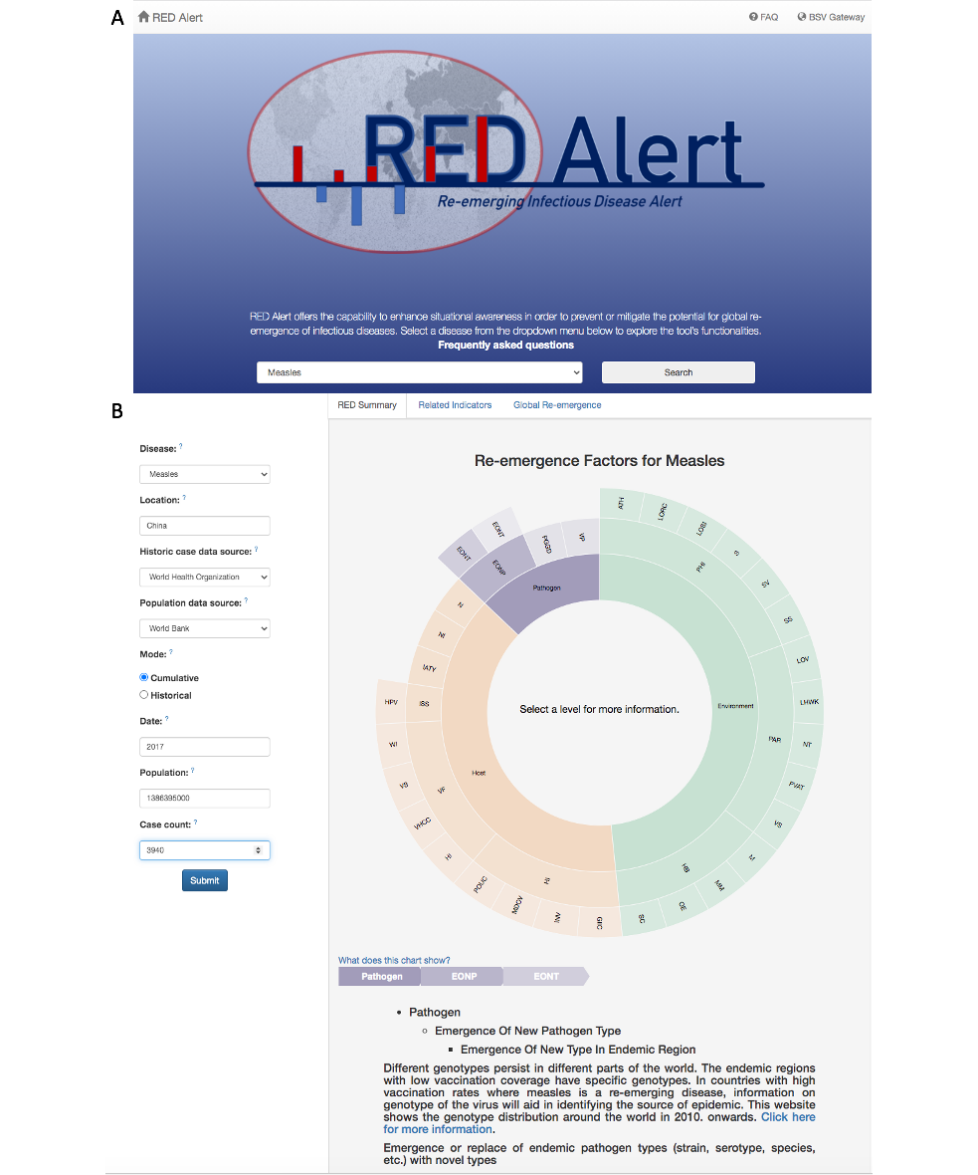
RED Alert’s input form and causal wheel. Panel A shows RED Alert’s first tab, which provides descriptions about the application’s core functionalities and allows user to select the disease of interest. Panel B shows the causal wheel for measles. It includes factors known to have contribute to resurgence of measles in previous scenarios. Factors at the center of circle represent components of epidemiological triad and expanding distance from the center of the circle correspond to increased specificity of factors contributing to component causes. When the user clicks on a component cause, detailed information about the component cause is provided at the bottom of the chart along with the associated references.

We developed a re-emergence causal wheel for each disease in RED Alert; an example can be seen in Figure 1B. The causal wheel was modeled on the epidemiological triad [43]: host, pathogen, and environment. In the causal wheel, these categories were further divided into subcategories based on disease-specific factors that contribute to re-emergence identified from the literature. We thus created multiple rings around the primary inner ring of the epidemiological triad in our visual display for this information in RED Alert. For example, for cholera, the broad category of environment was divided into socioeconomic and natural factors affecting the environment which included natural environment, population density, public health infrastructure, and human behavior. The natural environment was further divided into weather patterns, climate change, and natural disasters. Natural disasters were further divided into floods, typhoon/hurricane, earthquakes, and drought. This causal wheel is displayed on the web application when a user selects a disease of interest, providing general information about the component causes of re-emergence for the disease. We also added links to detailed information about a component cause to facilitate access.

These component causes were mapped to 1 or more indicator variables (obtained from the World Bank), which served as the proxy measurement for the corresponding component cause. Assessment of these component causes and their interactions can help guide effective intervention strategies. We developed a table for visualization of disease-specific indicators that allows comparison of the values for the user’s location and year of interest to the historical range, so that the user can determine which re-emergence cause and indicator might be contributing to country’s potential re-emergence.

Component causes and corresponding related indicators for a given disease and location from 2000 to the year of interest are shown in the table. If data were not available for the year of interest, the indicator value for the most recent year when the data are available was displayed. We also identify indicators where the values for the year of interest are outside the 25th and 75th percentile or 10th and 90th percentile, as these indicators show relatively extreme values for the re-emergence year as compared to the historical values for the location of interest and hence may be potential contributing factors for the disease re-emergence. These indicators and components are displayed to the user in a form of table along with the associated value for the year of interest and statistics for historically observed values (eg, median and 25th and 75th percentiles). Indicators with values outside the 10th and 90th percentiles are highlighted in dark red or dark blue colors if they are potential risk or protective factors, respectively. Similarly, indicators with less extreme values (ie, values outside the 25th and 75th percentiles) are highlighted in light red or light blue colors if they are potential risk or protective factors, respectively.

#### Understanding Potential for Global Disease Re-emergence

To help identify the potential for global re-emergence, we developed a visual summary in the form of a map showing the spatial distribution of national re-emergence events (identified through the machine learning classifier described above) worldwide within recent history (last 10 years). The map is time enabled, allowing the user to scroll dynamically through the last 10 years of historic data. Re-emergence events in the year selected by the slider are colored in red, whereas re-emergence events identified in previous years are identified by black points. The size of the points represents the number of historic re-emergence events in the last 10 years. Multiple re-emergence events across different countries or continents may suggest potential for global re-emergence and require further investigation by the user.

#### Additional Visual Analytics

In addition to features developed to answer the 3 primary objectives described above, we developed visual analytics that could help deepen the understanding of potential contributing factors for the re-emergence and global re-emergence assessment. RED Alert visual analytics were developed to illustrate the relationship between potential contributing factors (eg, sanitation facilities, urbanization, or vaccinated percentage of the population) and disease re-emergence. We also developed visual analytics to compare locations with similar disease incidence (ie, locations with incidence within 50%-150% of user-specified data). These additional analytics were provided in a second tab of the RED Alert output.

To help assess the global disease re-emergence situation, we organized different types of global data in a third tab of RED Alert. This includes information about disease incidence globally for the last 10 years from the year of interest input by a user and recent reports of disease occurrence on FluTrackers [48], an online disease community bulletin board. We also provided the following questions on this tab that guide users through the data and facilitate hypothesis generation:

- Are the highest 2 quantiles of disease incidence dispersed over multiple continents?
- Has disease incidence intensified, across geographic areas, over time?
- Are the most recent FluTrackers community posts dispersed over multiple continents?

### Evaluating RED Alert Through Case Studies

To evaluate the performance of the fully developed RED Alert analytic, we used case studies for each of the 4 diseases (measles, dengue, cholera, and yellow fever). Specific inputs were identified based on the outbreak selected, and we evaluated the output with respect to its utility in addressing the 3 main objectives that the visual analytic was developed for: (1) Can we identify potential disease re-emergence in a country? (2) What might be the contributing factors to re-emergence in that location? and (3) Are there indications of a global re-emergence based on the input situation? Using the same case studies, we also evaluated the utility of the additional visual analytics that guide hypothesis generation and provide actionable information to the user. We identified the scope of use and the type of actionable information that can be obtained from RED Alert by defining specific work roles to also understand the broad utility and diversity of information that can be used.

Case studies were selected from the 2015 to 2019 timeframe to best illustrate every feature of the analytic. One of the primary challenges is the availability of updated global data. As RED Alert is dependent upon the updating cycle of data sources used (World Bank and WHO), it is often difficult to examine all the features using the current year. Complete, global data sets for public health indicators and infectious disease case counts are currently available up to 2017 or 2018. However, we believe this is still a reasonable representation of situations that occurred in 2019/2020 and the near future of about 5 years, as the natural and built environments are not expected to significantly change in such a short timeframe.

## Results

### Detecting Potential Disease Re-emergence

We selected random forest as the classifier to integrate into RED Alert because it outperformed the decision tree classifier in terms of the F_2_ score for the re-emergence class for all diseases. Table 1 shows the performance of random forest classifiers in terms of average and SD of precision, recall, F_1_, and F_2_ measures over 10 nested cross-validations. For the specific diseases in RED Alert, the models were able to identify 82%-90% of all potential re-emergence events as potential re-emergence cases. Of all instances classified as potential re-emergence, about 19% to 31% (except 46% for dengue) were false positives. Our models identified most of the country-level re-emergence events identified in the literature while missing a few events that were restricted to smaller geographic areas and did not contribute enough disease cases to affect disease incidence at the country level. In some cases, our models also identified earlier disease re-emergence events as compared to the literature, underscoring the utility of our models for early detection and warning.

**Table 1:** Random forest performance over 10 nested cross-validation.^a^

Measure and class		Measles	Cholera	Dengue	Yellow fever
		Mean (SD)	Mean (SD)	Mean (SD)	Mean (SD)
**Precision**					
	RED	0.7100 (0.1015)	0.8100 (0.1197)	0.5411 (0.0436)	0.6914 (0.1270)
	Not RED	0.9925 (0.0057)	0.9913 (0.0063)	0.9883 (0.0040)	0.9964 (0.0036)
**Recall**					
	RED	0.9064 (0.0736)	0.8236 (0.1267)	0.8421 (0.0554)	0.8856 (0.1130)
	Not RED	0.9689 (0.0147)	0.9889 (0.0098)	0.9480 (0.0117)	0.9857 (0.0087)
**F_1_**					
	RED	0.7909 (0.0688)	0.8051 (0.0814)	0.6567 (0.0439)	0.7631 (0.0752)
	Not RED	0.9805 (0.0075)	0.9901 (0.0046)	0.9677 (0.0060)	0.9910 (0.0037)
**F_2_**					
	RED	0.8546 (0.0601)	0.8129 (0.0971)	0.7557 (0.0425)	0.8278 (0.0781)
	Not RED	0.9735 (0.0118)	0.9893 (0.0074)	0.9558 (0.0094)	0.9878 (0.0066)

^a^RED and not RED represent re-emergence and non-re-emergence classes, respectively.

### Evaluation of RED Alert Through Case Studies

RED Alert features 2 primary modes for users to engage with the application: cumulative and historical analysis. The modes depend on the user’s access to data and the user’s willingness to upload data into the application. It is important to note that any data the user inputs in the form is not stored by the application at any point. The lowest burden mode to the user is the historical mode. This mode displays all historic data as calculated incidence for the user’s defined location. The cumulative mode is of moderate complexity and is the most frequently utilized option in RED Alert. This mode requires that the users know the year they are interested in analyzing as well as the corresponding case counts. For each disease, the analytic provides the most appropriate data source depending on the location. A user selects the cumulative mode if he/she intends to utilize the tool to explore how the data relate to the historic collection of case counts. We describe the results of using RED Alert for a case study for measles. We describe additional case studies for yellow fever, cholera, and dengue in Multimedia Appendix 1. The tool is very rich in information and data, and wherever possible, we have tried to evaluate how the different facets of the analytic could support different types of analysis.

For the measles case study, we specified a public health analyst as the work role and identified the following task for the analyst: Determine the historical profile of measles in China over the past several decades to review the natural temporal fluctuations in measles, and determine if the reported case count for China in 2017 is indicative of a re-emergence. Following the selection of measles from the drop-down menu on the first tab (Figure 1A), the first image seen was a sunburst chart (Figure 1B) that provided the user information on the various causes of re-emergence of measles. The causes were broadly categorized into host, pathogen, and environmental causes, and the user could obtain further detailed information for each of these causes. For example, one of the pathogen-specific factors leading to re-emergence is a new measles type introduced into an endemic country. The following case study inputs were used to generate answers to the 3 main questions used for evaluating the tool: Location—“China,” population data source—Default World Bank), mode—cumulative, year of interest—2017, number of cases—3940.

**Figure 2:**
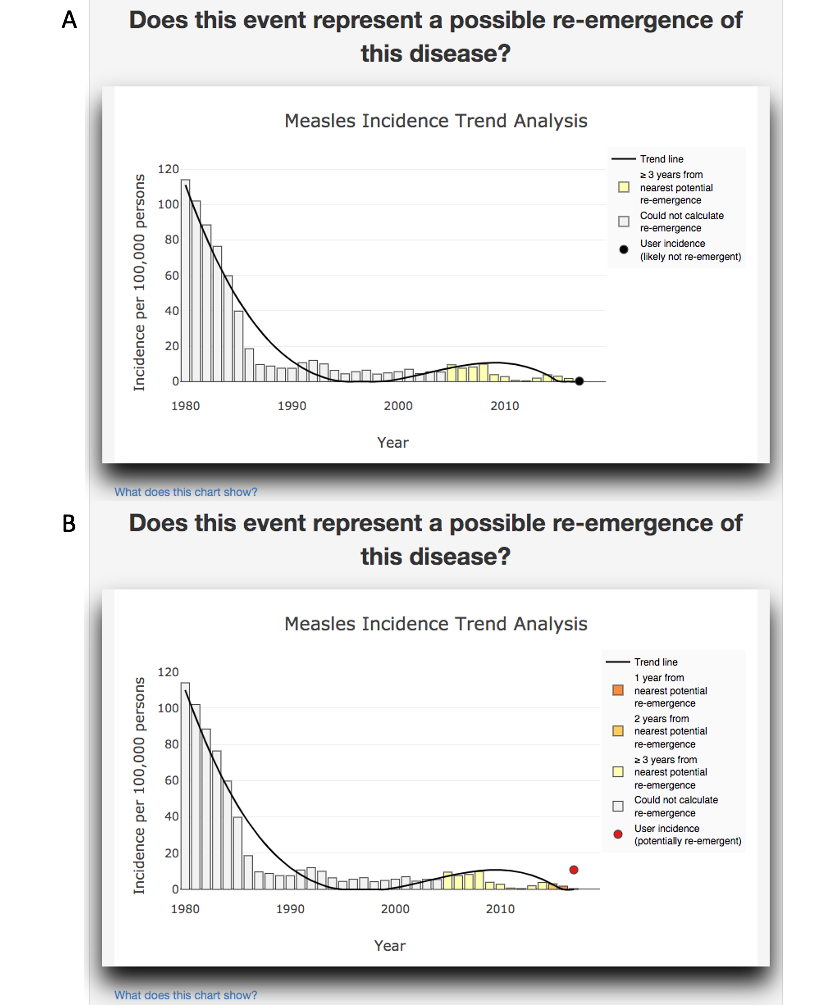
Measles incidence trend analysis and re-emergence detection for China. Panel A shows the case corresponding to 3940 measles cases in China in 2017 (as in the case study). The model does not predict re-emergence for this case. Panel B shows the case when the number of measles cases in China are increased to 150,000 in 2017. As this represents a large change in incidence, the model predicts potential re-emergence.

The output was seen in a tabbed format, with the first tab “RED summary” showing the answers to the 3 primary questions:

Q1, “Does this event represent a possible re-emergence of this disease?”: The time series (Figure 2A) showed a dip in incidence in 2011 and 2012 followed by a slight rise in cases in 2013 and 2014 and a steady decrease since then. The legend on the chart also indicated that the input data did not reflect a potential re-emergence. When the case count was changed to 150,000, the chart did change (Figure 2B) and the legend on the chart indicated a potential re-emergence together with a red dot on the chart.

**Figure 3:**
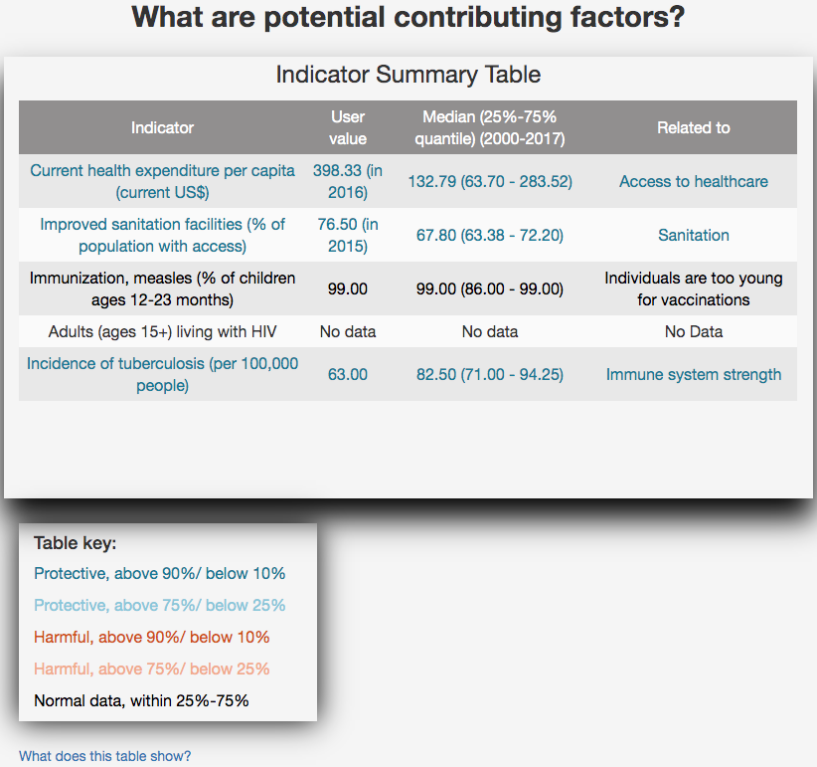
Potential contributing factors for measles in China.

Q2, “What are potential contributing factors?”: A summary table (Figure 3) showed the range of factors that potentially contribute to re-emergence, including the values of public health indicators that map to causes of RED for measles, for both the user input year and the median for the recent history (2000 to present). Harmful or protective values were colored red or blue.

**Figure 4:**
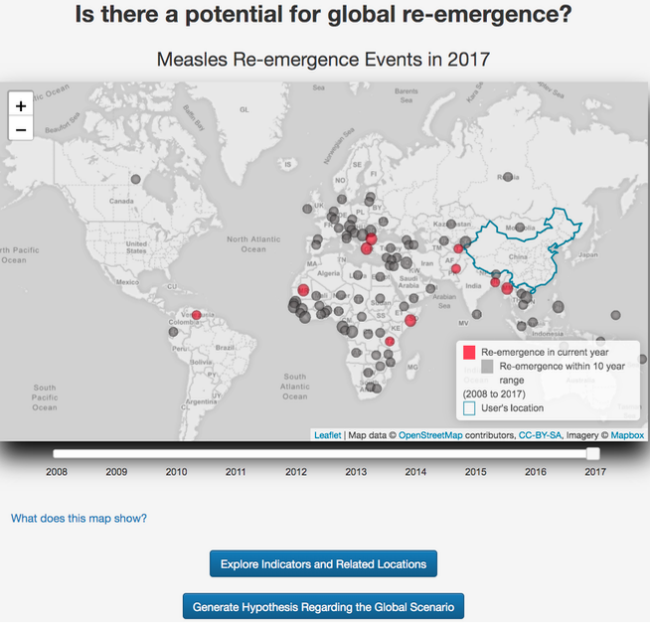
Distribution of national measles re-emergence events worldwide.

Q3, “Is there a potential for global re-emergence?”: A dynamic review of the past 10 years from input year (Figure 4) showed that global re-emergence likely began around the 2008-2009 timeframe. Interestingly, most experts identified global re-emergence around the 2011-2012 timeframe, indicating that RED Alert could have provided earlier warning. Within the past 5 years from 2017, several countries showed re-emergence of measles, but the geographic distribution was concentrated in Eastern Europe and Africa. Myanmar and Bangladesh, which border China, experienced potential re-emergence of measles in 2017, but the disease did not travel across the border to elicit a similar disease event in China.

Thus, RED Alert was able to successfully address the primary objectives for which it was developed, and provide actionable information.

**Figure 5:**
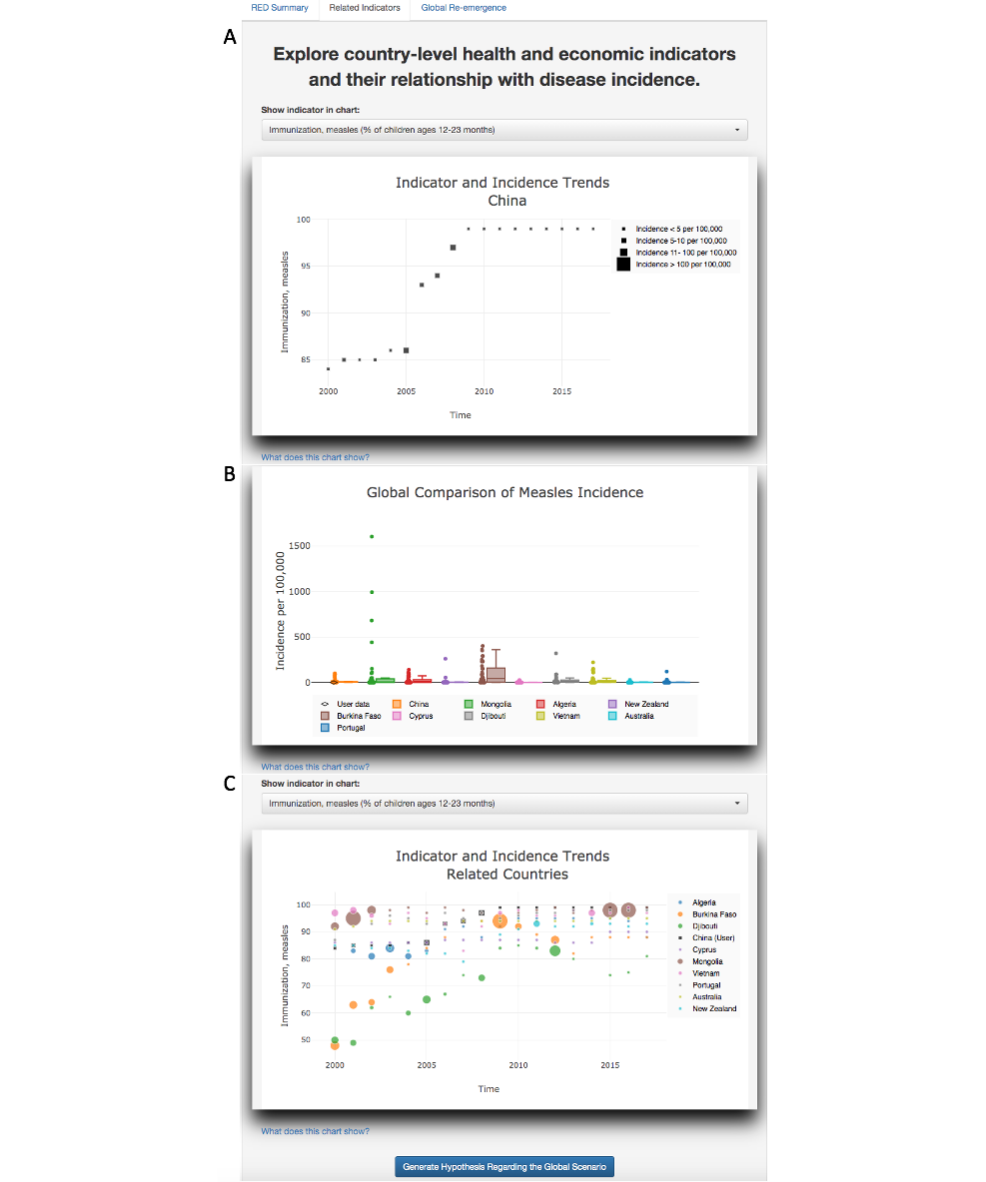
RED Alert’s related indicators tab. Panel A shows relationship between measles incidence and measles vaccination for China. Panel B shows global comparison of measles incidence. Panel C shows indicator and measles incidence trend for related countries.

The output of the additional analytics was examined on the “related indicators” tab. Selection of “Immunization, measles (% of children ages 12-23 months)” for the first plot (Figure 5A) on this tab showed that the measles immunization rate exceeded 90% and was maintained above the 90% threshold since 2006, offering a potential reason as to why re-emergence was not identified in China. The comparative boxplot (Figure 5B) showed the countries that had an incidence between 50% and 150% of China’s incidence in 2017, offering a global context. For example, the chart showed that New Zealand and China had very similar incidence perhaps due to similar vaccination rates. This hypothesis could be validated by the selection of “Immunization, measles (% of children ages 12-23 months)” above the third plot (Figure 5C), which showed incidence rates and vaccination coverage to be similar within the past 5 years for New Zealand and China.

**Figure 6:**
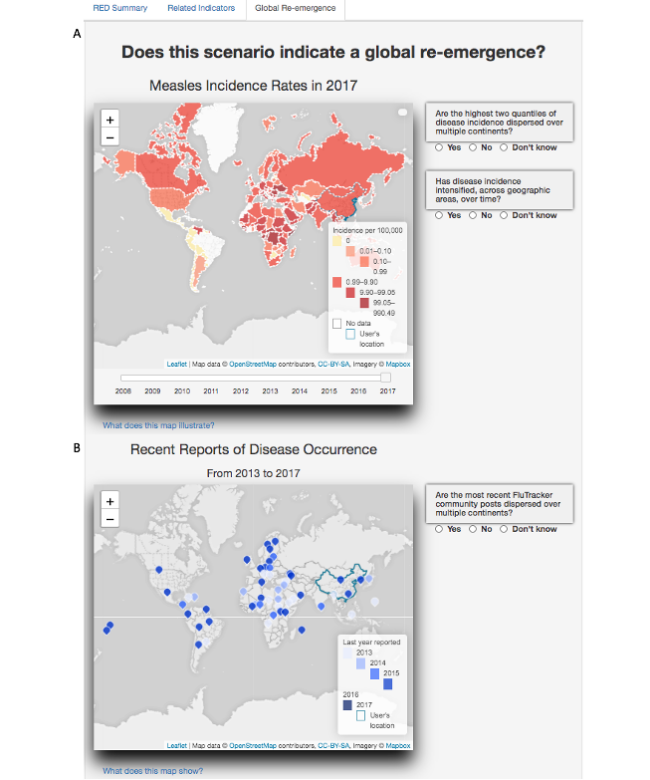
RED Alert’s global re-emergence tab. Panel A shows measles incidence worldwide. Panel B shows recent reports of disease occurrence for measles based on a disease bulletin.

Finally, the utility of visual analytics to understand the global scenario of re-emergence was examined on the “Global Re-emergence” tab. The first global map (Figure 6A) showed the incidence in 2017 and the highest incidence values in Africa. A dynamic review of the past 10 years showed that the incidence was globally higher 5 years before 2017. The second global map showed that measles had been discussed on the international disease bulletin website FluTrackers across all continents within the past 2 years (Figure 6B). These maps provided a context to the 2017 China situation and indicated that global re-emergence of measles has occurred much earlier.

## Discussion

### Principal Findings

In this paper, we presented RED Alert, a web-based tool that can provide early warning and detection of infectious disease re-emergence (not disease emergence). It is designed to help public health analysts detect and understand disease re-emergence at both the local (ie, country level) and the global scale through contextual data analysis. It uses supervised machine learning models to detect local disease re-emergence and visual data analytics to help identify and explore potential factors contributing to this re-emergence and assess global situation for potential disease re-emergence. Consistent with our goal of identifying all potential cases of disease re-emergence events while allowing some false positives, our supervised learning models were able to classify 82%-90% of the re-emergence cases, however, with 19% to 31% (except 46% for dengue) false positives. A detailed evaluation of the models used for re-emergence detection is described in [9]. We have also evaluated the utility of the tool through a number of case studies. RED Alert contains all the relevant information to not only provide early warning for potential re-emergence of disease locally and globally, but also offers causes for it. Through the diverse visual presentations and data at their fingertips, RED Alert allows users to verify their hypotheses about local and global re-emergence, and thus facilitates decision making in real-time. A user can access this tool as a one-stop shop for both data and relevant analyses and write a complete report.

While there are a number of online tools for disease surveillance [15,16,18], to the best of our knowledge, this is the first tool that is designed specifically for re-emerging diseases and focuses on detection of potential re-emergence at both local and global level as well as identification of potential contributing factors for the local re-emergence event.

Prior work in disease re-emergence has focused on the contributing factors of re-emergence. In particular, recent work has focused on the tremendous impact of climate change [49,50]. Changes to the climate impact almost every facet of disease transmission from increasing the habitat of disease vectors [51] to increasing the threat of civil unrest and violence [52], which in turn destabilizes infrastructure necessary for resiliency to re-emergence. To complicate this, it is clear that human factors such as urbanization and international travel also impact disease re-emergence [5-8]. However, despite the fact that the literature is clear that there is a complex system at work, the authors have not been able to find any other work in the data fusion or visualization space to allow public health experts to actually interact with the components necessary. Indeed, it is because of this complex milieu that RED Alert is necessary.

Our hope is that RED Alert can provide actionable information to public health analysts and decision makers that can be used for planning purposes. Our tool can provide indications that disease re-emergence may be occurring in a given region (or globally) and also help inform the user of possible contributing factors. This information may be useful in helping better understand the situation, as well as helping determine possible mitigations.

Currently, the tool has data for 4 diseases at the country level and yearly time scale. However, our methodology is applicable to other diseases, as well as other spatial and temporal scales. In addition, although the current application is designed for use on a laptop or desktop computer, we are currently also developing a mobile app for this tool.

### Limitations

RED Alert is the first tool designed for detecting and understanding disease re-emergence and provides novel analysis. However, it relies on the availability and quality of data, which depends upon the public health infrastructure of the country. Under-reporting is common in biosurveillance systems [53]. While there are some missing data, historical data collected by the tool are relatively complete. By contrast, there is often some delay in reporting case counts data to agencies such as the WHO or PAHO or data collection companies such as Gideon. Similarly, there is also some delay in the estimation and availability of population and related indicators on the World Bank website. This often leads to missing data for many countries for a couple of recent years. To deal with this, we allow users to input recent case counts data and use values from the latest available years for population and related indicators for analysis purpose. We believe this is reasonable, as this information is less likely to change significantly in a short period. However, discrepancies in the data may affect our analysis.

While it is common in machine learning applications for humans to label data, due to the lack of the concrete definition of re-emergence, labeling is a subjective assessment. It may be possible that the SMEs in our team mislabeled data in some cases. Further, due to the lack of a concrete quantitative definition of re-emergence, it is difficult to fully validate our analysis.

### Future Directions

There are many opportunities for future work including adding more diseases to the tool based on their likelihood of re-emerging. Currently, the ability to perform the same analysis at a subnational level is mainly restricted by data availability. We are working on obtaining data at subnational levels for a few diseases and countries and plan to make this functionality available through a mobile app. Re-emergence detection models can also be improved by using other disease-related factors such as weather or climate data for mosquito-borne diseases, as mosquito density depends upon temperature and humidity.

## Appendix

Multimedia Appendix 1 Additional case studies for cholera, dengue, and yellow fever to show utility of RED Alert in detecting and understanding disease re-emergence.

Multimedia Appendix 2 Data Sources for RED Alert.

## Abbreviations

API: application programming interface
PAHO: Pan American Health Organization
SME: subject matter expert
WHO: World Health Organization
