# Supplementary material for: Improving Detection of Disease Re-emergence Using a Web-Based Tool (RED Alert): Design and Case Analysis Study
Source: JMIR Public Health Surveill. 2021 Jan 7;7(1):e24132. doi: 10.2196/24132 (PMC7819778; doi:10.2196/24132)
Supplement: Multimedia Appendix 1 [file publichealth_v7i1e24132_app1.docx]

## Multimedia Appendix 1: Case Studies for RED Alert.

This document describes case studies for cholera, dengue, and yellow fever to illustrate the functionality of RED Alert. The reader is walked through all the features of the tool using the case study of measles in the main paper. For case studies of cholera, dengue, and yellow fever, we present outputs from the first summary tab that answers three key questions on re-emergence of the specific disease and relies on the algorithm used for the tool. Tabs 2 and 3 are meant for deeper dives by users into data accessed by RED Alert and facilitate hypothesis generation and testing.

**Cholera**

**Scenario**: In 2016, a military strategist in charge of operations wants to know if the recently observed case counts of cholera in Iran could present a threat to military personnel in the region.

The analyst lands on the home page of RED Alert and reads through the features available within the application. Finding the application a good fit for her needs, the user selects cholera from the drop-down and clicks search to investigate the current situation. The user explores the causal factors that are derived from the host and the environment nodes of the epidemiologic triangle by clicking through the re-emergence factors graphic (Figure 1). Interestingly, the region adjacent to the proposed deployment site has been experiencing a severe drought over the past two years, which corresponds with one of the environmental indicators of re-emergence. The user has been informed of 1,567 cholera cases that have been reported throughout the country. Exploring the input options, the user selects Iran as the location, and the tool auto-populates Gideon as the historical cases count data source and World Bank as the population data source. The user identifies the cumulative mode will best suit her investigation. The user enters 1,567 case counts and selects 2016 as the date.


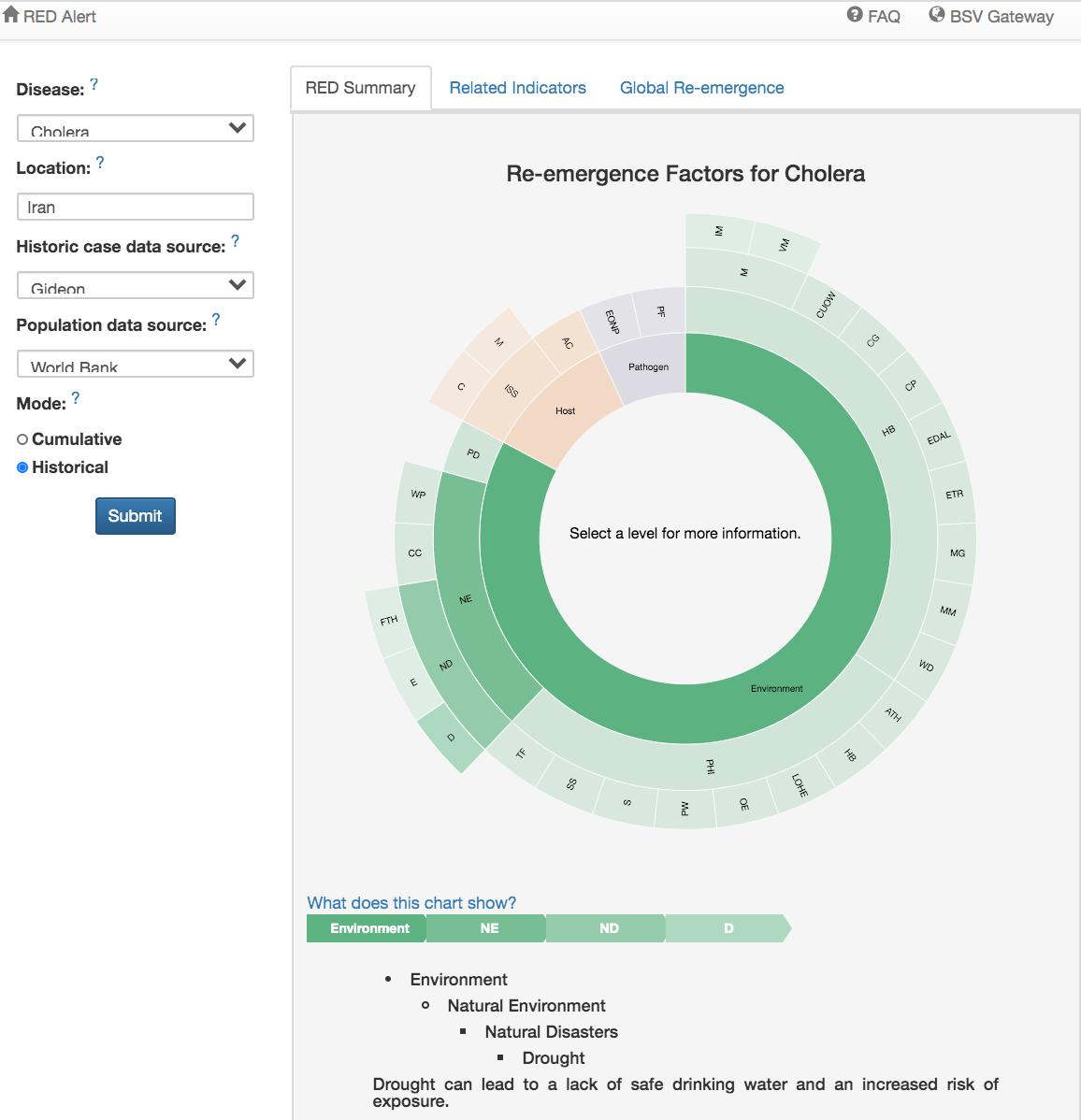

Figure 1. Causal Wheel for cholera. This shows factors known to have contribute to resurgence of cholera in previous scenarios. Factors at the center of circle represent components of epidemiological triad and expanding distance from the center of the circle correspond to increased specificity of factors contributing to component causes.


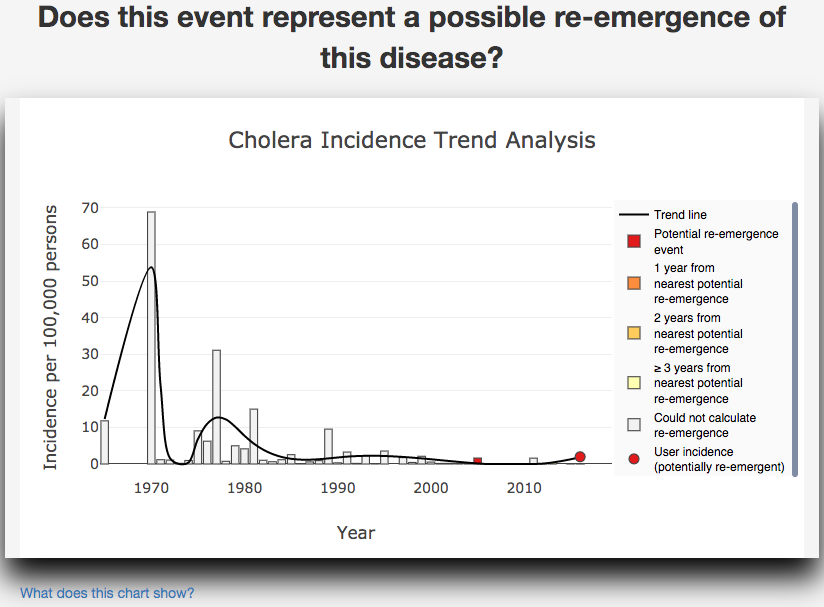


Figure 2. Cholera incidence trend analysis and re-emergence detection for Iran.


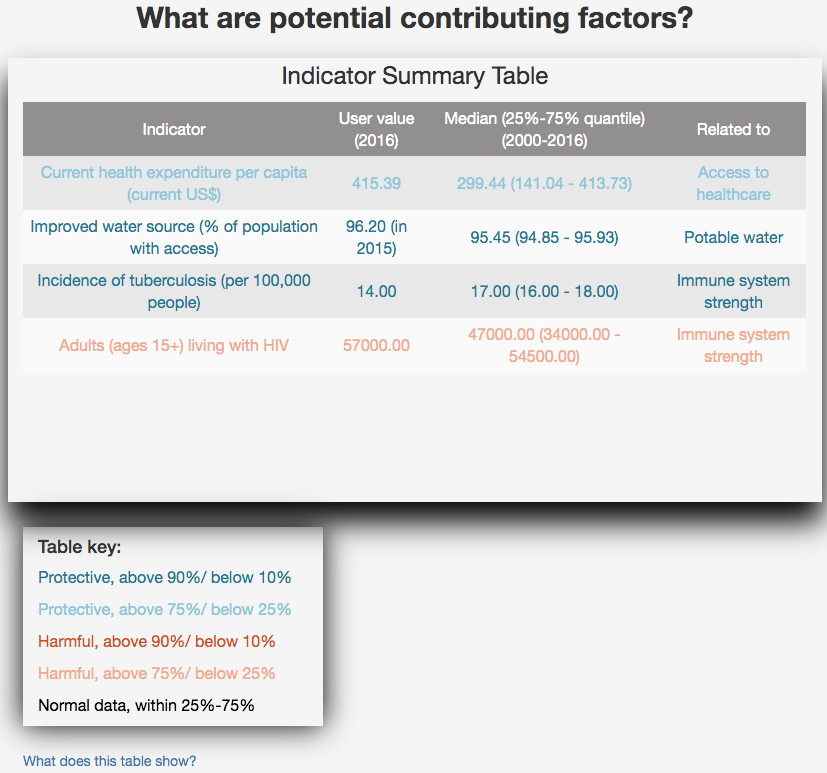


Figure 3. Potential contributing factors for cholera in Iran. This table shows a summary of indicators related to the component causes outlined in the causal wheel. Indicators with extreme current values (i.e., values outside 10th and 90th percentiles of historical values) are highlighted in dark red or dark blue colors. Similarly, indicators with less extreme current values (i.e., values outside 25th and 75th percentiles) are

highlighted in light red or light blue colors. Here, blue and red colors indicate whether the indicator has protective or harmful effect on disease incidence, respectively. If data are not available for the year of interest, then the indicator value for the most recent year when the data are available is extracted.

**
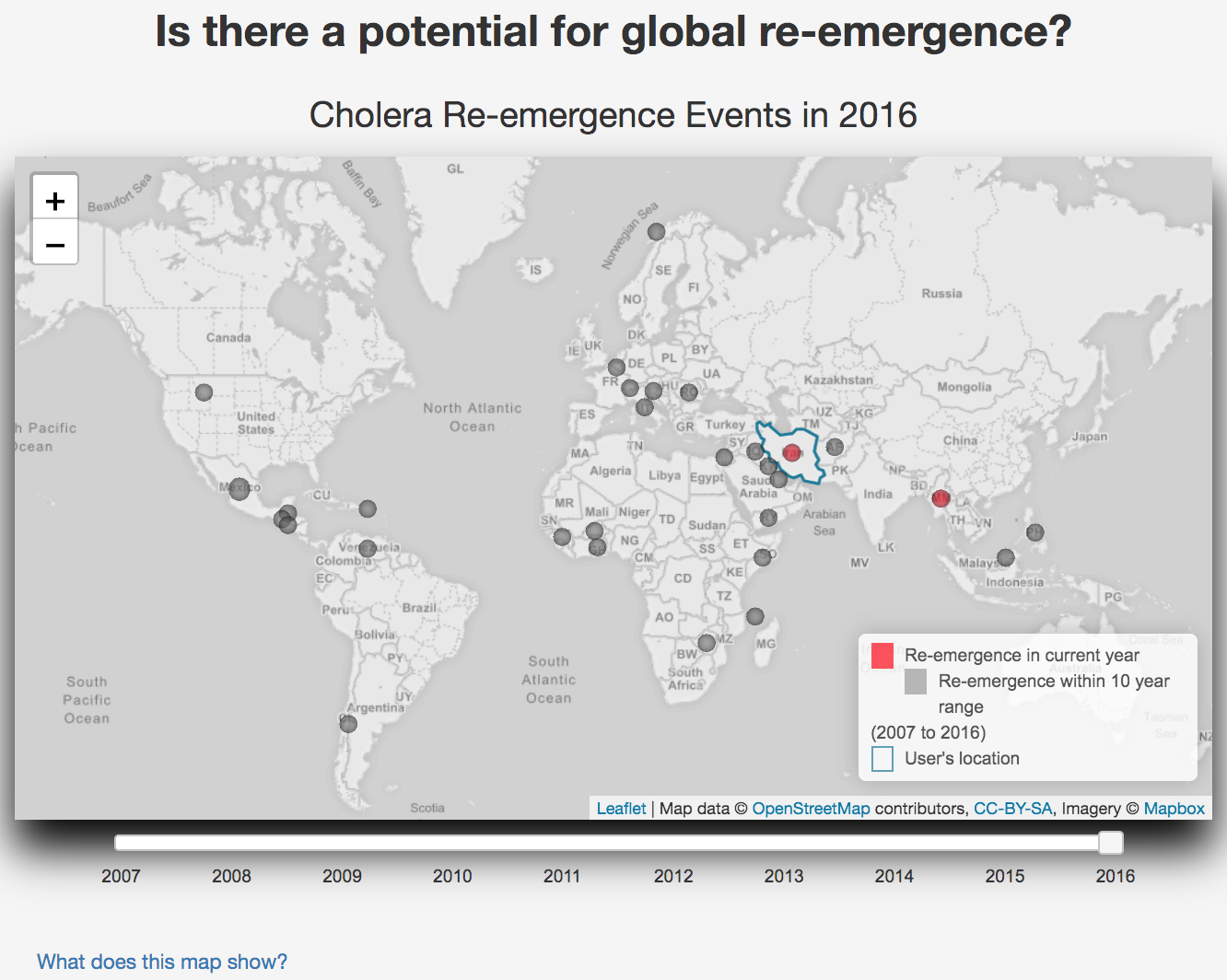
**

Figure 4. Distribution of national cholera re-emergence events worldwide.

The summary chart (Figure 2) displays the user's calculated incidence is 1.97 and the point is colored in red. The user references the legend and observes that the case count she had entered is likely to represent a disease re-emergence event. The user exports this graph to her report. The user finds that the summary table (as shown in Figure 3) shows data for the available indicators with the last available year. The user observes that the indicator of Adults (ages 15+) living with HIV could potentially be a cause of disease re-emergence, given its value over the global range. The user then views the map at the bottom of the page (as shown in Figure 4) and observes that one other re-emergent event was identified in 2016 in Southeast Asia. Using the slider, the user observes that the national cholera re-emergence events throughout the world are relatively low compared to earlier years, but she also notes that some neighbors of Iran may have had recent re-emergences, namely, Bahrain in 2015 and Afghanistan in 2014. Armed with this information, the strategist is able to generate a report that will inform the preparations that must be made for troops to be deployed in that area.

**Mosquito-borne Diseases, Dengue and Yellow Fever**

**Scenario**: A health operations manager at the WHO with substantial experience in mosquito borne diseases is performing routine surveillance for dengue in Philippines in 2019 and yellow fever in Brazil in 2018. She wants to know if this years is defined as re-emergence for the disease/location pair, and what the general patterns of disease incidence have been over the past decade or so. She has received information about this country from the Ministries of Health and realizes that she could access RED Alert for this analysis.

**Dengue**

The analyst knows that the number of cases reported by Philippines department of health as of August 2019 is 167,606, the highest in the last 5 years. The user accesses RED Alert from Los Alamos National Laboratory's BSV Gateway (https://redalert.bsvgateway.org) and after reading the brief descriptions he selects dengue from the drop-down menu. The user selects Philippines as the location and clicks the cumulative mode radio button. The tool auto-populates the data sources to be used for the historical case counts (Gideon), historic population for Philippines (World Bank) and the current population. The user fills in selects a year and enters case counts. The tool indicates that as the 2019 population is not available, the last updated information from 2018 will be used as shown in Figure 5. This feature has been in included in the tool to provide transparency on what data is being used for our algorithms. Accepting this notification, he submits the form. The user observes that the first chart (as shown in Figure 6) indicates the occurrence of two potential re-emergence events, over the past two decades with the last one occurring in 2010. Thereafter there has been a steadily increasing trend of cases and while the computed user incidence for 2019 is fairly high, it is on the upward slope of the chart and not identified as a new re-emergence event. This is very useful information given the media hype about the current outbreak. A look at the summary table (Figure 7) that identifies cause for potential re-emergence shows several indicators for Philippines that are in the harmful category. These relate to an increase in an immunocompromised population and increase in population density, which together likely contribute to faster spread of dengue and rising cases. These indicators map back to both host and environmental factors for disease re-emergence. These two charts help the user understand that dengue has already re-emerged so it is urgent that mitigation plans are put in place and long term strategies developed for mosquito control.


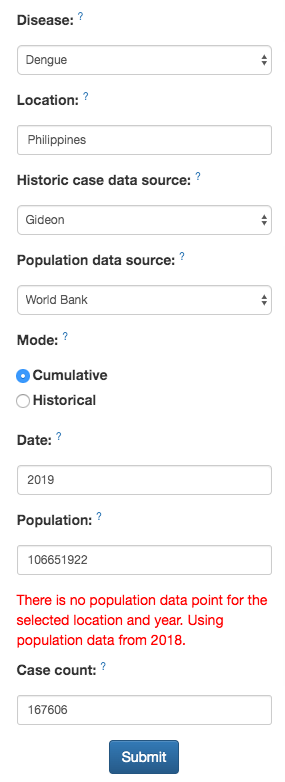


Figure 5. User input fields for RED Alert.


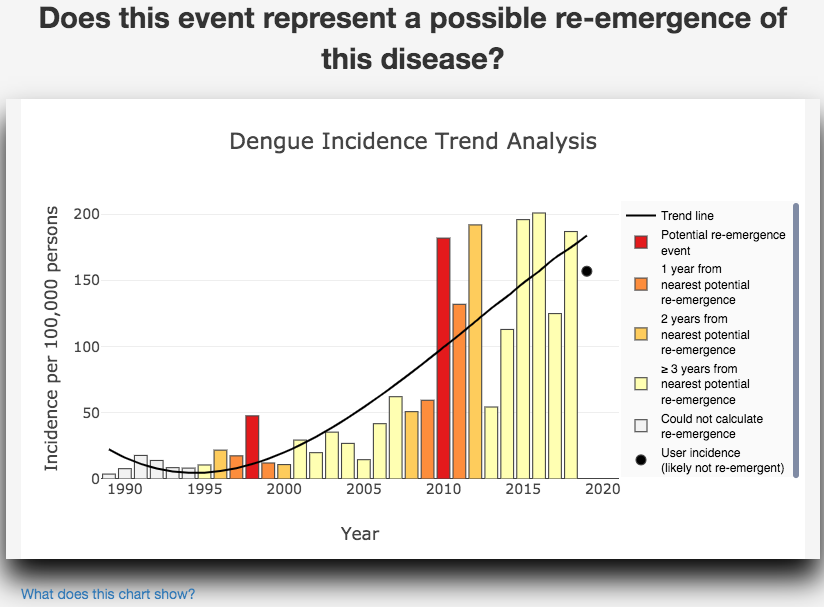


Figure 6. Dengue incidence trend analysis and re-emergence detection for Philippines.

**
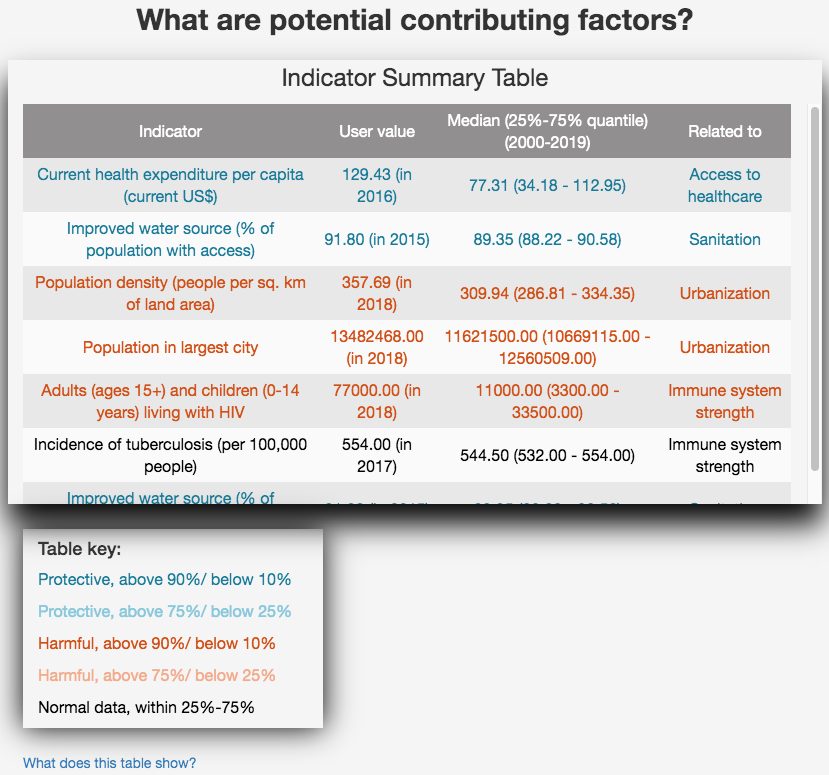
**

Figure 7. Potential contributing factors for dengue in Philippines.

**Yellow Fever**

The analyst is aware from WHO reports that there was a spike in yellow fever cases in 2018 (https://www.who.int/csr/don/18-april-2019-yellow-fever-brazil/en/), approaching 1,400. This case count was higher than previous years and she wants to find out when/if yellow fever re-emerged in Brazil. Her hypothesis is that the rise in disease incidence seen in 2018 is actually on the upward slope of a disease that re-emerged two or more years prior. She submits the relevant case count, year, and location information and reviews the output for Brazil. As can be seen from the Figure 8, there seem to have been multiple waves of yellow fever re-emergence, and as the analyst suspected, the current rise in disease incidence is reflective of potential re-emergence that occurred in 2015 (nearest potential re-emergence event). The user's point is being identified as not re-emergent because the event has already occurred. An examination of the potential causes for re-emergence in Brazil (Figure 9) indicate environmental factors such as increase in population in the largest city and population density being prominent which likely contribute to faster spread of yellow fever. The analyst reviews the global re-emergence chart and using the slider, the user observes that following the re-emergence event in Brazil in 2015 (Figure 10, Panel A), the analytic shows potential re-emergences occurring in neighboring countries of Colombia (Figure 10, Panel B), Ecuador and Bolivia (Figure 10, Panel C) in subsequent years. This is important information for the analyst. She is able to get a perspective of the global scenario rapidly using the RED Alert tool.


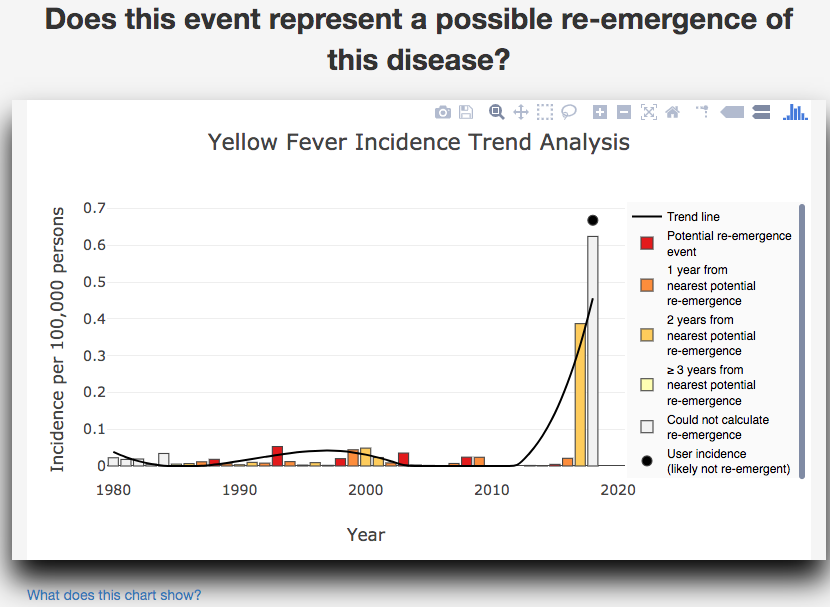


Figure 8. Yellow fever incidence trend analysis and re-emergence detection for Brazil. This graph provides information about historical disease incidence values, overall trend of incidence over time (illustrated using spline to smooth incidence fluctuations), and current as well as historical re-emergence events for Brazil.

####
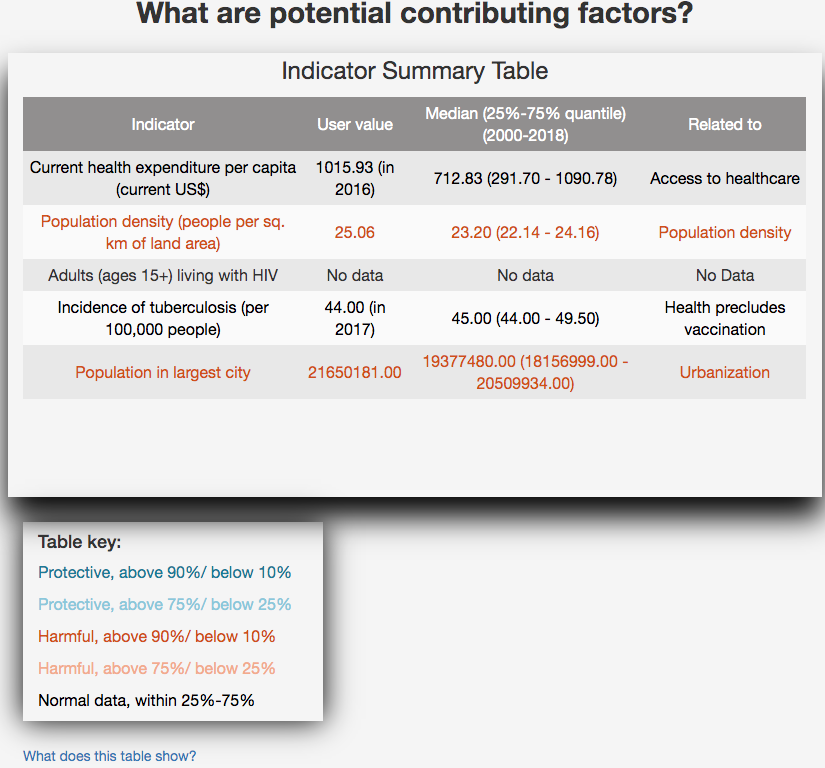


Figure 9. Potential contributing factors for yellow fever in Brazil. Indicators with extreme current values (i.e., values outside 10th and 90th percentiles of historical values) are highlighted in dark red or dark blue colors. Similarly, indicators with less extreme current values (i.e., values outside 25th and 75th percentiles) are highlighted in light red or light blue colors. Here, blue and red colors indicate whether the indicator has protective or harmful effect on disease incidence, respectively. Examination of the indicators in this table suggests that increase in population of the largest city and population density may be the factors contributing to the faster spread of yellow fever in Brazil.


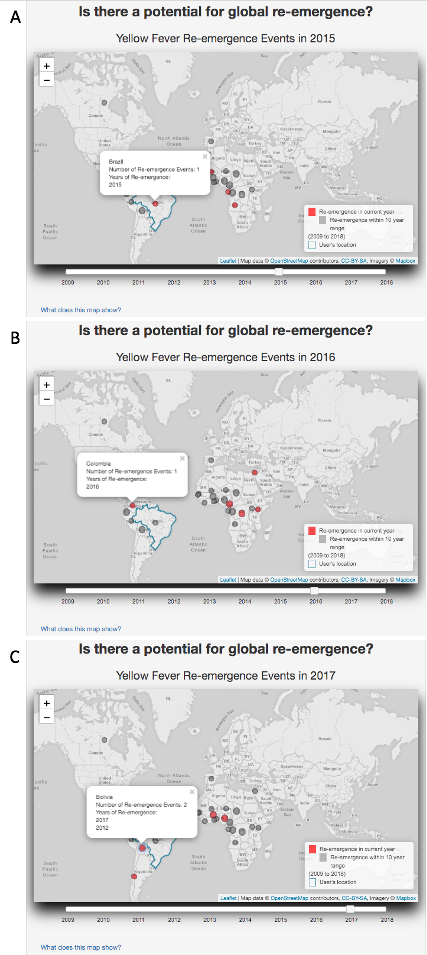


Figure 10. Distribution of national yellow fever re-emergence events worldwide. Panels A, B, C and show national yellow fever re-emergence events for 2015, 2016, and 2017.
