# Supplementary material for: Improving Detection of Disease Re-emergence Using a Web-Based Tool (RED Alert): Design and Case Analysis Study
Source: JMIR Public Health Surveill. 2021 Jan 7;7(1):e24132. doi: 10.2196/24132 (PMC7819778; doi:10.2196/24132)
Supplement: Multimedia Appendix 2 [file publichealth_v7i1e24132_app2.docx]

## Multimedia Appendix 2: Data Sources for RED Alert.

This document described data sources used for RED Alert. It focused on four diseases: measles, cholera, dengue, and yellow fever. The models used to detect re-emergence use features based on case counts, population, WHO region, and disease-specific features such as vaccination coverage for measles and number of severe case counts and deaths for dengue. We also use data from World Bank Indicators to identify factors that potentially contribute to disease re-emergence.

## Case Counts

Historical case count data, together with sub categories like severe dengue and deaths are obtained from the WHO [1-3], Gideon [4], and the Pan American Health Organization (PAHO) [5]. For each disease, the tool provides the most appropriate data source depending on the location.

## Population

We collect population data from two datasets: LandScan [6] and World Bank population data [7]. LandScan [6] provides an ambient population (average over 24 hours) estimate at approximately 1km^2^ spatial resolution for each year starting from 1998 (except 1999). The total population for each location (i.e., city, state, and country) is obtained by overlaying location boundary polygons over the LandScan raster and summing populations for all cells within the boundary polygon. Population data from the World Bank provides mid-year population estimates at the country level from 1960 to 2017. While we primarily use World Bank population data in the tool, we provide the option for a user to compute disease incidence using LandScan data. It is our intention to expand this tool for utility at subnational scales (admin level 1), where LandScan data will become more useful.

## Vaccination Coverage for Measles

The World Health Organization [8] provides vaccination coverage information at the country-level (for all the countries where cases were reported) for each year from 1997-2018 and at every five years from 1980 to 1995. We obtained rates for Measles-containing-vaccine first-dose (MCV1) and second-dose (MCV2).

## Related Indicators

Host, pathogen, and environment represent the traditional epidemiological triad [9] and can provide information about the potential factors that contribute to re-emergence. To identify indicators that can be a proxy for re-emergence causes and hence related to potential re-emergence, we collect indicator data from the World Bank [10]. We downloaded data from the following topics using the World Bank application programming interface (API) [11]: World Development Indicators, Education Statistics, Gender Statistics, Health Nutrition and Population Statistics, Millennium Development Goals, Health Nutrition and Population Statistics by Wealth Quintile, Global Financial Development, Doing Business, Worldwide Governance Indicators, Enterprise Surveys, Sustainable Development Goals, Sustainable Energy for All, and Wealth Accounting.

## WHO Regions

WHO member countries are grouped into six regions [12]. For each region, we obtained the list of countries by following region-specific links. For countries in our database that did not belong to any of the WHO regions, we assigned one using neighboring countries when reasonable using the WHO region map available at [45].

## References

1. World Health Organization vaccine-preventable diseases: monitoring system. 2017 global summary. http://apps.who.int/immunization_monitoring/globalsummary.
2. World Health Organization, Global Health Observatory data repository, Number of reported cases Data by country. http://apps.who.int/gho/data/node.main.175?lang=en.
3. World Health Organization, DengueNet, Welcome to the DengueNet database and geographic information system. http://apps.who.int/globalatlas/default.asp.
4. Gideon. https://www.gideononline.com/.
5. PAHO. https://www.paho.org/data/index.php/en/mnu-topics/indicadores-dengue-en/

dengue-nacional-en/257-dengue-casos-muertes-pais-ano-en.html.

1. Oak Ridge National Laboratory, LandScan™ . https://landscan.ornl.gov/.
2. The World Bank, DataBank | Population estimates and projections. http://databank.worldbank.org/data/reports.aspx?source=population-estimates-and-projections.
3. World Health Organization Measles-containing vaccine. http://apps.who.int/immunization_monitoring/globalsummary/timeseries/tscoveragemcv1.html.
4. Principles of epidemiology in public health practice: an introduction to applied epidemiology and biostatistics. U.S. Departmentt of Health and Human Services, Centers for Disease Control and Prevention (CDC), Office of Workforce and Career Development; 2012.
5. The World Bank, DataBank | Data | Indicators. https://data.worldbank.org/indicator.
6. The World Bank, DataBank | Data | About the Indicators API Documentation. https://datahelpdesk.worldbank.org/knowledgebase/articles/889392-apidocumentation.
7. World Health Organization | About WHO | WHO regional offices. http://origin.who.int/about/regions/en/.
